# Fatal pulmonary embolism following shoulder arthroplasty: a case report

**DOI:** 10.4076/1752-1947-3-8708

**Published:** 2009-07-31

**Authors:** Thayur R Madhusudhan, Sanath K Shetty, Savitha Madhusudhan, Amit Sinha

**Affiliations:** 1 Department of Trauma and Orthopaedics, Glan Clwyd hospital, Rhyl, LL18 5UJ, UK; 2 Department of Ophthalmology, H M Stanley Hospital, St. Asaph, LL170RS, UK

## Abstract

**Introduction:**

Fatal pulmonary embolism following a shoulder joint replacement is a rare event. The exact prevalence of shoulder arthroplasties is not clear. Unlike hip and knee joint replacements where some form of thromboprophylaxis is routinely prescribed, no such guidelines and practice exist for shoulder replacements. Other case reports have confirmed fatal and non-fatal pulmonary embolisms following shoulder replacements, but particular risk factors were identifiable in those patients.

**Case presentation:**

We report the case of a 73-year-old Caucasian woman with fatal pulmonary embolism secondary to a calf deep vein thrombosis following a shoulder joint replacement procedure. The patient was otherwise healthy; there were no other risk factors directly contributing to deep vein thrombosis and pulmonary embolism except for a body mass index of 34. Post-mortem examination confirmed that the patient had a thrombus in the calf and a pulmonary embolus.

**Conclusions:**

Fatal deep vein thrombosis and pulmonary embolism can occur following shoulder joint replacements in otherwise normal patients. A high degree of suspicion should therefore be maintained in susceptible individuals. Thromboprophylaxis needs careful consideration in shoulder replacements in susceptible individuals.

## Introduction

Deep vein thrombosis and pulmonary embolism (DVT/PE) are recognized complications of hip and knee joint replacements. Because definite guidelines in addressing these conditions are already in place, some form of thromboprophylaxis is routinely prescribed to high risk patients. DVT/PE occurrences, however, are uncommon in shoulder arthroplasties and there are no guidelines currently in place. Case reports and other recent studies do suggest, however, that these events are no longer as rare as suggested in the literature. The physician needs to consider these events in shoulder replacements.

## Case presentation

A 73-year-old Caucasian woman was admitted for elective shoulder replacement for an arthritic shoulder. She was being treated for hypertension with beta-blockers and thiazide diuretics and her blood pressure was well controlled. She had a history of diverticulitis and varicose vein surgery with an uneventful outcome. Her body mass index (BMI) was 34 during pre-operative anesthetic evaluation. She had no family history of DVT/PE. Blood investigations including electrolytes, liver function tests and coagulation screening were normal. Results of her electrocardiogram (ECG) and chest X-ray were unremarkable. The comorbidities were well optimized and she was admitted a day before her operation.

The patient was operated under general anesthesia supplemented with an interscalene block for postoperative analgesia. An intermittent calf compression device was used for mechanical thromboprophylaxis intraoperatively. The operation was performed through an anterior deltopectoral approach. The patient had no intraoperative complications. The patient recovered satisfactorily from general anesthesia and was monitored in a high dependency care unit for postoperative optimization. The operated shoulder was supported in an arm sling support for comfort. The patient was prescribed postoperative opioid analgesics and antibiotic prophylaxis with three doses of cefuroxime. No chemical or mechanical thromboprophylaxis were used postoperatively.

The patient was already feeling well on the next postoperative day and her blood parameters were satisfactory. Her pain was well controlled and her arm continued to be immobilized by the sling. There was no excessive oozing from the surgical wound and there were no distal neurovascular deficits. The patient's calves were soft and nontender and she was ambulating well postoperatively. A systemic examination of the patient was unremarkable. However, while being monitored she suddenly became breathless, hypoxic and hypotensive and experienced intense sweating. She did not complain of chest pain and an ECG was negative for cardiac ischemic changes. Her PaO_2_ was 84% on room air. She was resuscitated with oxygen by mask and initiated on low molecular weight heparin in a therapeutic dose. Despite all resuscitative measures the patient could not be revived. A post-mortem examination was requested, which confirmed pulmonary embolism and left calf deep vein thrombosis.

## Discussion

Deep vein thrombosis and pulmonary embolism are recognized complications of hip and knee joint replacements and pelvic operations. Definite guidelines for prevention and treatment exist [1] in knee and hip arthroplasties. Earlier studies reported that the occurrence of pulmonary embolism is a rare event following shoulder arthroplasty [2,3] and that a high degree of suspicion is warranted should the patient develop respiratory difficulty following a shoulder arthroplasty. There were no reported fatal events related to such conditions.

However, recent studies [4,5] have indicated that although the absolute rates of thromboembolic complications were lower in patients who had shoulder arthroplasties compared with those who had lower limb procedures, a larger percentage of these complications were pulmonary embolisms. Perioperative antithrombotic prophylaxis may be beneficial to reduce the frequency of deep venous thrombosis and pulmonary embolism among patients undergoing shoulder arthroplasties, particularly in higher-risk groups. A relatively high incidence of deep vein thrombosis after shoulder arthroplasty has also been reported. Still, more studies are required for definite conclusions and for establishing guidelines for thromboprophylaxis.

Saleem et al. report that the origin of thrombus in their reported patient was secondary to a deep vein leg thrombosis and this was attributed to a period of prolonged immobilization in the perioperative period [6]. In our patient, there were no identified risk factors for DVT or PE during pre-anesthetic evaluation. Clinical examination in the postoperative period was normal, and the patient was ambulating well with no signs of deep vein thrombosis in her calf. Due to a lack of clinical evidence and an absence of definite risk factors, no chemical and mechanical thromboprophylaxis was considered postoperatively. An intermittent mechanical compression device used intraoperatively may not prove beneficial in this setting. For the same reason, we were also unable to correlate the association between deep vein thrombosis of the legs and shoulder arthroplasty in our patient. Further exploration may point to other less known risk factors that could have contributed to the process of venous thrombosis and the subsequent pulmonary embolism.

## Conclusions

Deep vein thrombosis and pulmonary embolism can occur and prove fatal following shoulder arthroplasty. Thromboprophylaxis needs careful consideration in the selection and management of patients.

## Abbreviations

BMI: body mass index; DVT: deep vein thrombosis; ECG: electrocardiogram; PaO_2_: Partial Pressure of Oxygen in Arterial Blood; PE: pulmonary embolism.

## Consent

Written informed consent was obtained from the next of kin of the patient for publication of this case report. A copy of the written consent is available for review by the Editor-in-Chief of this journal.

## Competing interests

The authors declare that they have no competing interests.

## Authors' contributions

TRM was the principal author and was involved in the collection of data, review of literature, and preparation of the manuscript. SKS and SM were involved in the collection of literature and in editing the manuscript. AS was the senior author and was actively involved in patient care and in editing the manuscript. All authors read and approved the manuscript.
